# Hiatal hernia involving prolapse of the entire stomach into the mediastinum after distal gastrectomy: a case report

**DOI:** 10.1186/s40792-018-0503-7

**Published:** 2018-08-13

**Authors:** Takuro Konno-Kumagai, Daisuke Takeyama, Toru Nakano, Tadashi Sakurai, Yusuke Taniyama, Takahiro Heishi, Chiaki Sato, Takashi Kamei

**Affiliations:** Division of Advanced Surgical Science and Technology, Graduate School of Medicine, University of Tohoku, 1-1 Seiryo-machi, Aoba-ku, Sendai, Miyagi 980-8574 Japan

**Keywords:** Large hiatal hernia, Postgastrectomy, Fundoplication

## Abstract

**Background:**

Prolapse of a small part of the proximal stomach through the hiatus into the mediastinum is relatively common. Hiatal hernia involving the postoperative stomach has been reported previously, but the degree of hernia was not so severe, and hiatal hernia involving the prolapse of the entire stomach following gastrectomy into the mediastinum has never been reported. We describe a very rare case of large hiatal hernia involving the entire postoperative stomach.

**Case presentation:**

A 79-year-old man with a history of distal gastrectomy for submucosal benign tumor 40 years ago was referred to our hospital because of dysphagia and weight loss. Computed tomography revealed prolapse of the entire postoperative stomach into the mediastinum, and a radical operation was performed. There was a strong adhesion in the hernial sac of the mediastinum, but only little adhesion due to a previous open surgery in the abdominal cavity was present. After the stomach was pulled into the abdominal cavity, suture cruroplasty and Toupet fundoplication without dissection of the short gastric artery were performed. The patient experienced postoperative paralytic ileus, but the rest of the postoperative course was uneventful and the symptom of dysphagia improved.

**Conclusions:**

We presented a very rare large hiatal hernia involving the entire postoperative stomach. Toupet fundoplication preserving the short gastric artery could be one of the optimal surgeries to prevent postoperative regurgitation of the remnant stomach.

## Background

Hiatal hernia is a type of diaphragmatic hernia characterized by the prolapse of the stomach or abdominal organs sliding through the hiatal orifice into the mediastinum or the thorax. Cases of prolapse of a small part of the stomach into the mediastinum without clinical symptoms are frequently experienced clinically, but cases of large hiatal hernia involving prolapse of other organs, such as the pancreas [1], colon [2], and entire stomach [3], into the mediastinum are relatively rare. Moreover, hiatal hernia involving the entire postoperative stomach has not been previously reported. Following distal gastrectomy, most of the arteries to the stomach are dissected except for the short gastric artery. Dissection of the short gastric artery is sometimes needed for fundoplication; therefore, assessment of the remaining blood flow to the remnant stomach by dynamic computed tomography (CT) and previous operative record is needed. We herein present a rare case of large hiatal hernia involving prolapse of the postoperative stomach into the mediastinum.

## Case presentation

A 79-year-old man was admitted to our hospital with symptoms of dysphagia and body weight loss of 10 kg for the past 3 months. His height was 158.0 cm and weight was 53.6 kg. His body mass index (BMI) was 21.5 upon admission. He was bent over due to osteoporosis and had undergone distal gastrectomy due to submucosal tumor (SMT) 40 years ago. According to his operative note, distal gastrectomy by open surgery was performed without lymph node dissection. After the retroperitoneal attachments behind the duodenum were dissected (Kocher maneuver), Billroth I reconstruction was performed. The resected mass was microscopically determined to be aberrant pancreas.

**Fig. 1:**
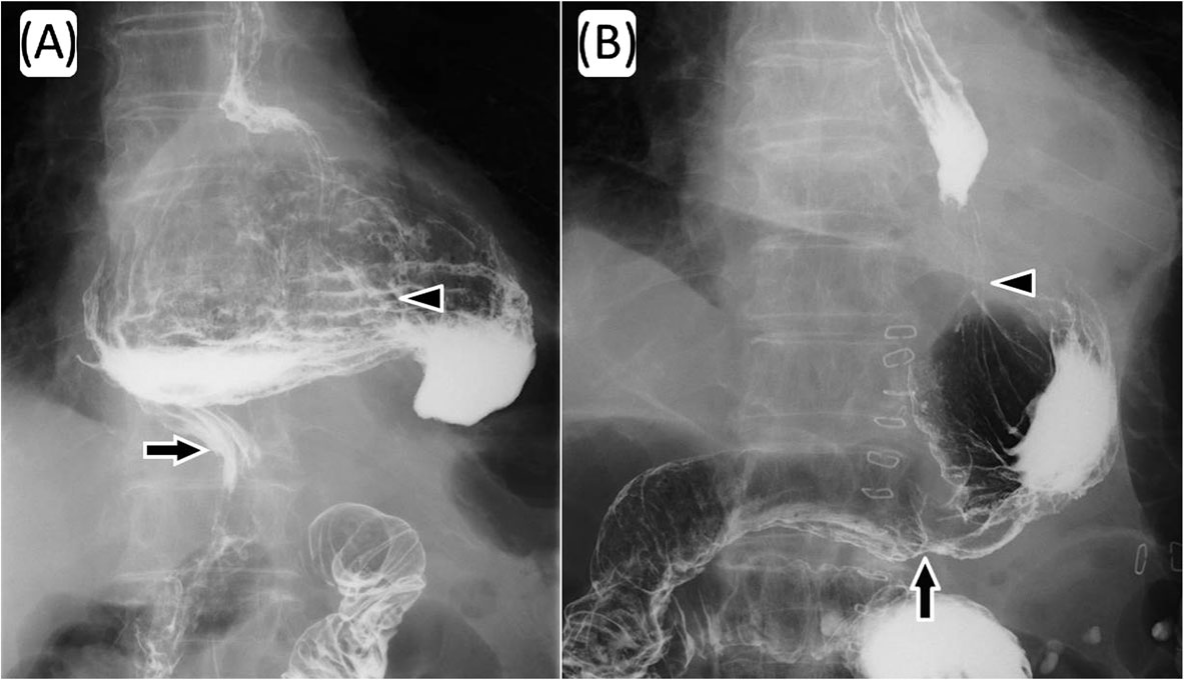
Upper gastrointestinal examination. The intrathoracic postoperative stomach with delayed barium passage was observed (**a**). Postoperative examination on day 2 after repair of the hiatal hernia shows the stomach located in the normal position and the absence of any obstruction and reflux of barium through the gastroesophageal junction (**b**). Arrow heads indicate gastroesophageal junction and arrows indicate gastroduodenal anastomotic site

**Fig. 2:**
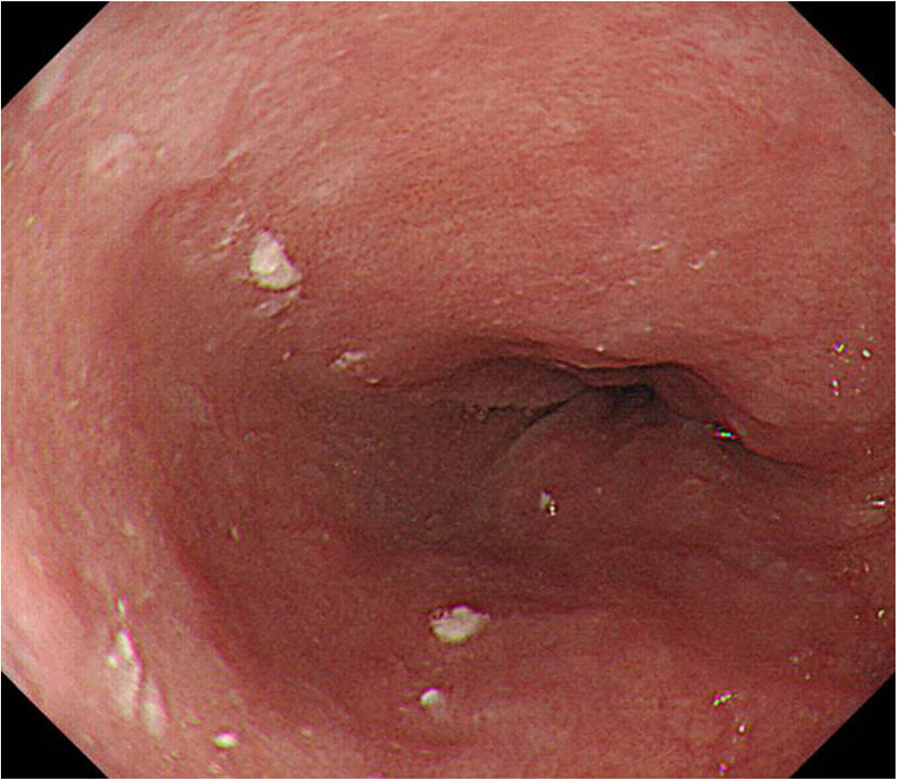
Upper endoscopic examination. Esophagitis was not found in gastroesophageal junction. There is no tumor in the upper digestive tract

**Fig. 3:**
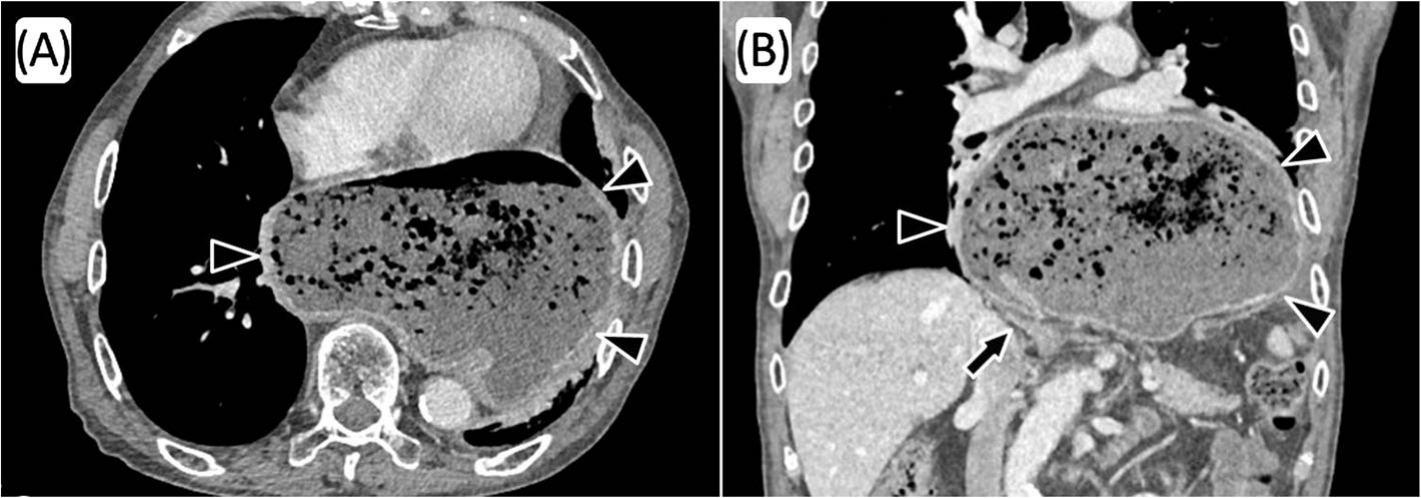
Contrast-enhanced computed tomography. A large hiatal hernia with the entire stomach (arrow heads) incarcerated through the hiatal orifice into the mediastinum, and the stomach was expanded with food remaining; in axial view (**a**) and in coronal view (**b**)

**Fig. 4:**
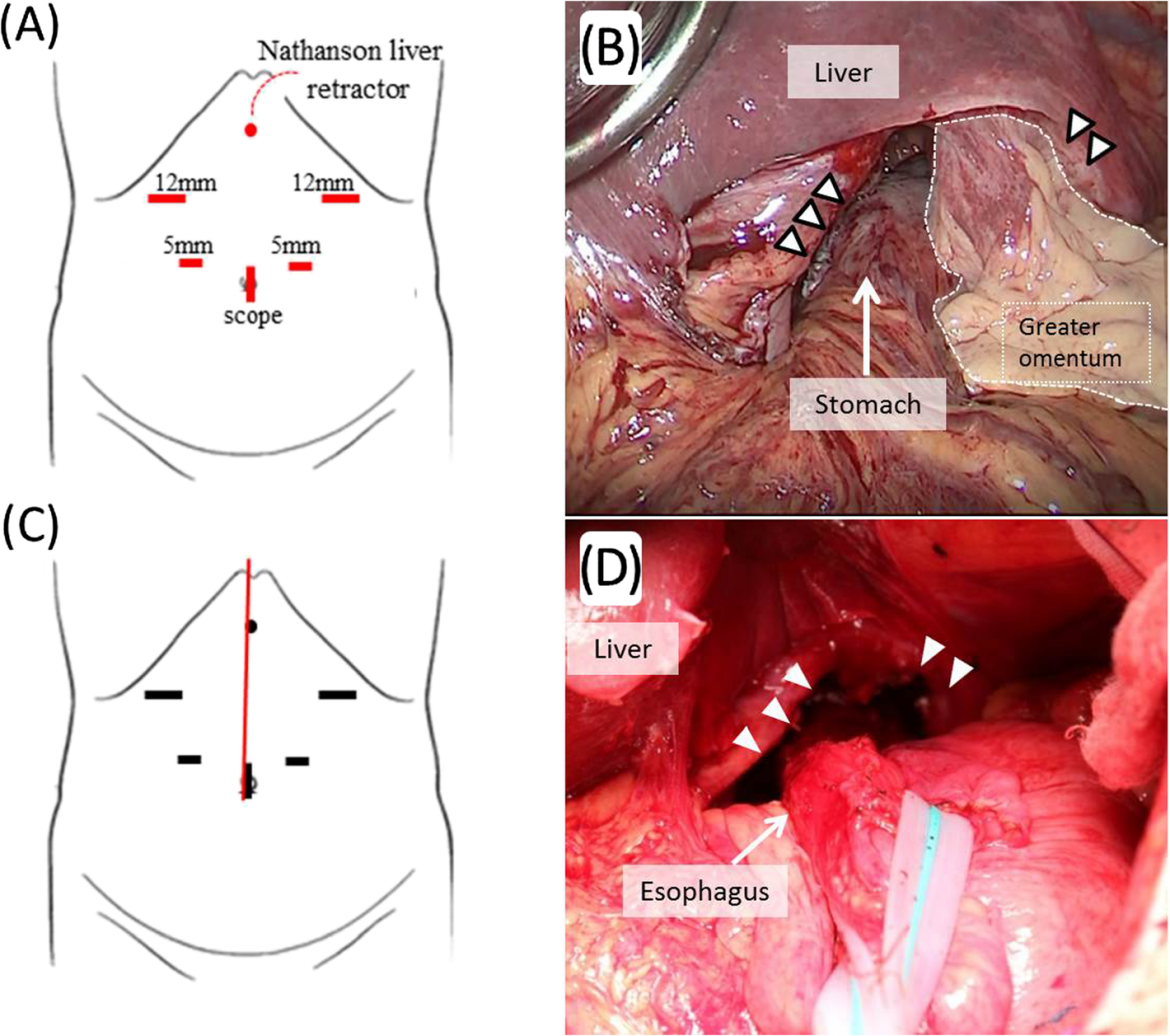
Port setting for hernia repair and operative findings. In total, five ports and Nathanson liver retractor were placed in the abdomen (**a**). A loose adhesion was observed just under the operative scar in the abdominal cavity; in contrast, the adhesion in the hernial sac was stronger. The esophageal hiatus was dilated to 7 cm in diameter (arrow heads). The hiatal hernia was identified and found to contain the entire postoperative stomach (**b**). We converted laparoscopic surgery to open surgery, and upper midline abdominal incision was added (**c**, red line). After manual reposition of the herniated content (**d**), cruroplasty with non-absorbable 2–0 sutures and Toupet fundoplication were performed

He was admitted to our hospital for dysphagia, and upper gastrointestinal examination revealed the presence of postoperative stomach in the thoracic cavity via delayed barium passage (Fig. 1a). Endoscopic examination showed that esophagitis and tumor in the upper digestive tract were absent (Fig. 2). Enhanced computed tomography (eCT) revealed a large hiatal hernia involving the entire stomach, sliding through the hiatal orifice into the mediastinum, and that the stomach was expanded with food remaining inside (Fig. 3a and b). The stomach in the posterior mediastinum compressed the heart; however, the patient had no symptoms of cardiac failure, and the heart function was normal on echocardiography. Preoperative eCT also showed that the right and left gastric arteries and the gastroepiploic arteries were preserved, indicating that the distal gastrectomy had been performed without lymph node dissection. He was diagnosed with hiatal hernia clinically, and the symptom of dysphagia was relatively severe; therefore, elective surgical repair of the hiatal hernia was performed. A total of five ports were placed in the abdomen, and a Nathanson liver retractor to retract the left liver lobe was inserted by blunt force (Fig. 4aa). A laparoscopic approach revealed a slight adhesion of the greater omentum just under the postoperative scar. Moreover, there was a little adhesion with the liver, spleen, and diaphragm in the abdominal cavity despite gastrectomy. The esophageal hiatus was dilated to 7 cm in diameter. The hiatal hernia was found to contain the entire postoperative stomach (Fig. 4b). The adhesion in the hernial sac of the mediastinum was stronger than that in the abdominal cavity, and safely exposing the stomach from the hernial sac was difficult. Therefore, we converted the laparoscopic surgery to open surgery, and an upper midline abdominal incision was added (Fig. 4c). After the dissection of the peritoneal hernial sac from the posterior mediastinum and manual repositioning of the herniated content (Fig. 4d), cruroplasty through interrupted sutures using non-absorbable 2-0 suture and Toupet fundoplication without short gastric artery division were performed. The operation time was 238 min, and intraoperative blood loss was 380 mL. Upper gastrointestinal examination on day 2 after the repair of the hiatal hernia showed that the stomach was located in the abdomen, with no obstruction or barium reflux through the gastroesophageal junction (Fig. 1b). Oral intake was started on the ninth postoperative day due to paralytic ileus after surgery. On day 15, after the repair of the hiatal hernia, he was discharged from the hospital without any symptoms of dysphagia. No dysphagia or recurrence of the hernia has been observed for 12 months after the surgery.

## Discussion

The following are the three common forms of esophageal hiatal hernia: sliding hiatal hernia, paraesophageal hernia, and combined type [4–6]. Hiatal hernia containing other abdominal organs were also reported, but these are rare [1, 2]. When abdominal organs prolapse into the mediastinum, which sometimes causes strangulation ileus or heart failure, surgical repair is often performed to reposition the organs [2, 7]. Prolapse of the proximal portion of the stomach through the hiatus into the thorax is relatively common; meanwhile, most of these hernias are small and produce few symptoms. However, in this case, the remaining postoperative stomach, estimated to be a third or half of the normal size, had prolapsed into the mediastinum. In addition, the esophageal hiatus was dilated to 7 cm in diameter, which is well in the range of a large hiatal hernia [8, 9], and was categorized as combined-type hiatal hernia. Sodeyama et al. [10] reported 39 cases of hiatal hernia following distal gastrectomy for gastric carcinoma or gastric ulcer, and they only found slight hiatal hernia, with a part of the proximal stomach being pulled into the mediastinum; hiatal hernia containing the entire postoperative stomach similar to our case has not been previously reported.

In general cases of hiatal hernia, aging is considered to be the cause [11]. The phrenoesophageal membrane, preaortic fascia, and median arcuate ligament are known as the structures fixing the gastroesophageal junction. When their ligaments become loose and the muscles of the hiatal tunnel widen due to aging, the risk of developing hiatal hernias would increase [11]. Esophageal hernia is exacerbated by positive intra-abdominal and negative intrathoracic pressures, which results in a large hernia [12]. Obesity is considered as one of the risk factors, and people who are bent over (gibbus deformity) because of aging may also have elevated intra-abdominal pressure. Moreover, gibbus deformity shortens the length between the esophageal hiatus and the duodenum, which could cause hiatal hernia. In the current case, although the patient’s BMI was not very high, the ligaments fixing the gastroesophageal junction may have loosened due to the aging process and the gibbus deformity may have increased the risk of hiatal hernia.

When gastrectomy is performed for gastric carcinoma, lymph node dissection is also performed routinely. Moreover, regions with lymph node dissection tend to have stronger adhesion with other organs around the stomach, such as the liver, spleen, and pancreas. Postoperative stomach also tends to adhere with other abdominal organs; therefore, prolapse of the postoperative stomach through the esophageal hiatus into the mediastinum seems rare. Sodeyama et al. [10] reported that the incidence of hiatal hernia was 37.5% in patients who underwent gastrectomy for gastric cancer or gastric ulcer, but the hiatal hernia cases they reported were not very serious. We performed a literature search in PubMed using “postoperative stomach” and “hiatal hernia” as keywords since 1990, and we did not find reports of large hiatal hernia after distal gastrectomy. To the best of our knowledge, we describe the first case of a large hiatal hernia involving prolapse of the entire postoperative stomach. One of possible cause of this rare case may be the gastrectomy performed for the benign tumor. As SMT, such as benign tumor or gastrointestinal stromal tumor, has few lymph node metastases, regional lymph node dissection is not usually performed, and according to the operative note of this case, the SMT on the antrum was dissected without involving the lymph nodes. In addition, dissection of retroperitoneal attachments behind the duodenum (Kocher maneuver) could have also caused the hiatal hernia, by shortening the length between the gastroesophageal junction and the duodenum, which is peculiar in Billroth I anastomosis reconstruction. Considering that the same surgical procedure without lymph node dissection is generally performed for gastrointestinal stromal tumor or benign SMT, more reports of large postoperative hiatal hernia, as in our case, should be reported. Therefore, the definitive pathogenesis of hiatal hernia similar to our case remains unclear.

Synthetic materials, such as mesh, are used to repair large esophageal hiatal hernia to reduce the risk of hernia recurrence. The meta-analysis by Furnée and Hazebroek [13] reportedly favored mesh cruroplasty over suture with regard to recurrence (26.3% after suture and 14.6% after mesh cruroplasty), whereas the meta-analysis by Tam et al. [14] did not. According to SAGES guideline, the use of mesh for reinforcement of large hiatal hernia repairs leads to decreased short-term recurrence rate, but there are not enough long-term data on which to base a recommendation either for or against the use of mesh [15]. Mesh-related complications, such as local erosion into the esophagus and esophageal stenosis, which is thought to cause higher dysphagia rates, are reported [3]. In this case, the esophageal hiatus was enlarged to 7 cm diameter, but it was relatively firm in the intraoperative finding. Therefore, only suture cruroplasty was performed to avoid mesh-related complications.

Laparoscopic repair with fundoplication in elective surgeries is currently the preferred procedure for patients with hiatal hernia. In addition, Furnée et al. [16] reported that the omission of an antireflux procedure for hiatal hernia, in the absence of preoperative symptoms due to gastroesophageal reflux disease, induced esophagitis in 28% of their patients. Sodeyama et al. [10] and Fujiwara et al. [17] previously reported that endoscopic esophagitis and reflux symptoms were found after distal gastrectomy in 20.2–39.5% and 27.9–65.8% of the patients. Therefore, we considered that the addition of an antireflux fundoplication was needed. Nissen (posterior total) and Toupet (posterior partial) fundoplications are currently common surgical approaches. Broeders et al. [18] reported that Toupet fundoplication reduces postoperative complications, such as dysphagia, compared with Nissen fundoplication. Taking into account the patient’s age, as aspiration pneumonia caused by dysphagia could be serious, Toupet fundoplication was selected in this case. Catarci et al. [19] reported that routine division of short gastric arteries during total fundoplication showed no significant advantages regarding the incidence of postoperative dysphagia when compared with no division. When there is tension around the abdominal esophagus, the procedure of dividing the gastrosplenic ligament, including the short gastric arteries, is sometimes needed to wrap the abdominal esophagus without obstruction to the passage of food. Therefore, addition of Toupet or Dor fundoplication, in which dissection of the short gastric artery is not necessarily required, would be a useful procedure for hiatal hernia of the postoperative stomach. In this case, the gastric and gastroepiploic arteries had been preserved, as revealed by preoperative eCT and operative findings; therefore, the short gastric artery could be dissected. However, the arteries to the stomach, except the short gastric artery, are often dissected in distal gastrectomy; therefore, Toupet repair preserving the short gastric artery could be one of the options for fundoplication of the remnant stomach.

## Conclusions

This is the first case report of a large hiatal hernia involving prolapse of the entire postoperative stomach into the thoracic cavity. Toupet fundoplication preserving the short gastric artery could be one of the optimal surgeries to prevent postoperative regurgitation of the remnant stomach.

## Abbreviations

BMI: Body mass index
CT: Computed tomography
eCT: Enhanced computed tomography
SMT: Submucosal tumor
